# Evaluating the Impact of Pre-Fermentative and Post-Fermentative Vinification Technologies on Bioactive Compounds and Antioxidant Activity of Teran Red Wine By-Products

**DOI:** 10.3390/foods13213493

**Published:** 2024-10-31

**Authors:** Sanja Radeka, Fumica Orbanić, Sara Rossi, Ena Bestulić, Ivana Horvat, Anita Silvana Ilak Peršurić, Igor Lukić, Tomislav Plavša, Marijan Bubola, Ana Jeromel

**Affiliations:** 1 Institute of Agriculture and Tourism, Karla Huguesa 8, 52440 Poreč, Croatia; sanja@iptpo.hr (S.R.); sarar@iptpo.hr (S.R.); ena@iptpo.hr (E.B.); ihorvat@iptpo.hr (I.H.); anita@iptpo.hr (A.S.I.P.); igor@iptpo.hr (I.L.); tomislav@iptpo.hr (T.P.); marijan@iptpo.hr (M.B.); 2 Department of Viticulture and Enology, Faculty of Agriculture, University of Zagreb, Svetošimunska Cesta 25, 10000 Zagreb, Croatia; amajdak@agr.hr

**Keywords:** wine by-products, pre-fermentative technologies, post-fermentative technologies, bioactive compounds, antioxidant activity, Teran red grape variety

## Abstract

This study aimed to evaluate bioactive properties of Teran red wine by-products (grape skins, seeds, and wine lees) from six vinification treatments, including a control (7-day standard maceration). Pre-fermentative cryomaceration (8 °C; 48 h) and hot maceration (50 °C; 48 h), followed by the 13-day (CS15; C15; H15) and 28-day (C30; H30) period, considering fermentation/maceration and extended post-fermentative maceration, were conducted. In CS15, the saignée procedure was applied before fermentation/maceration. After maceration, the separation of by-products was performed, followed by lyophilization and solid–liquid extraction. Then, individual phenols were analyzed using high-performance liquid chromatography (HPLC), and total phenolic content (TPC) and antioxidant activity (FRAP) were analyzed using UV/Vis spectrophotometry. The results showed grape skins and wine lees in all treatments had significantly increased TPC and FRAP values compared to the control. The highest concentration of total phenols (HPLC) in grape skins was found in CS15, at 978.54 mg/100 g DW. In wine lees, the highest concentration of total phenols was detected in the 30-day maceration treatments, at 582.04 mg/100 g DW in C30, and 595.83 mg/100 g DW in H30, despite the pre-fermentative procedure. In grape seeds, the highest concentration of total phenols was found in the control (K7), at 432.42 mg/100 g DW. Pre-fermentative heating together with extended 30-day maceration (H30) strongly reduced the total levels of phenols (HPLC and TPC) in grape seed samples. The findings implied an evident impact of pre- and post-fermentative technologies on phenols and antioxidant activity in wine by-products of *cv*. Teran (*Vitis vinifera* L.).

## 1. Introduction

Grapes are one of the most widely cultivated crops, primarily used in wine production [1,2]. They are considered a rich source of phytochemicals, which are partially extracted into wine during winemaking [3]. Thirty percent of the total quantity of vinified grapes correspond to wine by-products, which represents nearly 20 million tons per year globally, with around 50% of these by-products originating from the European Union [4]. The primary bio-waste products from the winemaking industry are grape stalks, grape pomace, and wine lees [5,6], of which the latter two are mostly reused in the food industry [4,7]. Grape pomace, the residue obtained after the pressing of red and white grapes/mashes in production of must or wine, is composed of skins, seeds, and in some cases, stems. During the vinification process, a substantial amount of grape pomace is generated, with a valuable recovery potential due to its significant content of valuable bioactive compounds such as phenols, mainly flavonoids, phenolic acids, and stilbenes [4,8,9]. In addition to phenols, these by-products also contain vitamins, proteins, lipids, minerals, carbohydrates, and other compounds including fibers [2]. Another notable by-product of vinification are wine lees, obtained as the solid deposits formed at the bottom of the tanks. These deposits mainly consist of yeasts and bacteria, carbohydrates, polyphenols, lignin, proteins, metals, salts, and organic acids [4,10].

Grapes and wines, rich in phenolic antioxidants, offer health benefits such as reducing oxidative stress, modulating inflammation, and preventing LDL oxidation [11,12]. Despite the high levels of antioxidant phytochemicals in winery by-products and advancements in extraction techniques, significant amounts of winery waste are still disposed of globally [6,13,14], leading to environmental issues like soil erosion and water pollution [2]. To mitigate these impacts, there is a growing need to shift from conventional methods to sustainable circular approaches that valorize waste through eco-friendly processes. The concept of a “circular bioeconomy” involves reusing waste materials produced during bioprocesses to create additional products [2,15]. Vinification by-products hold substantial potential for generating diverse bioproducts in agriculture, cosmetics, pharmaceuticals, biorefineries, and the food industry, including antimicrobial compounds, food additives, and nutraceuticals [2,4].

Since wine is the primary product obtained from grapes, extensive studies have been conducted to determine the impact of winemaking practices on the extraction of phenolic compounds into wine during winemaking. The application of various enzymatic preparations during grape fermentation/maceration facilitated the release of biologically active compounds from the solid grape components, leading to an increase in their concentration in the wine [16]. Additionally, to obtain high-quality wine, the optimization of the extraction through the application of either cooling or heating in various stages has been employed. Many pre-fermentative maceration procedures involve physical or chemical changes the grape tissue structure, thereby enhancing the release of desired compounds or increasing the yield of must [17]. These technologies include pre-fermentative mash cooling, commonly referred to as cold maceration, cold soaking, or cryomaceration. More commonly applied in winemaking, this technique improves quality characteristics such as wine color and aroma [18]. Cryomaceration refers to the skin contact with must in the absence of alcohol, allowing for the selective extraction of water-soluble compounds from the grapes. Both the temperature and the length of cryomaceration vary significantly, typically from 3 to 10 °C and lasting from several hours to several days, depending on the grape variety [19,20,21,22,23]. Conversely, when whole or crushed grapes are subjected to heating prior to alcoholic fermentation in order to produce musts or wines rich in color, the process is known as pre-fermentative heating or hot pre-fermentative maceration [22]. Heating causes the degradation and disruption of grape skin cell walls [24], facilitating the release of water-soluble phenolic substances [25]. The temperature during pre-fermentative heating generally ranges from 40 to 80 °C, with maceration lasting between 12 and 24 h or more, depending on the temperature [19,22,23,24]. Pre-fermentation juice runoff, i.e., the saignée procedure, is the procedure in which a portion of juice is removed before fermentation, resulting in a higher skin to juice ratio. This procedure theoretically boosts the concentration of anthocyanins and tannins in the final wine, since they are primarily located in grape skins and seeds [19]. The prolonged contact of grape mash and wine promotes the greater extraction of phenols, particularly catechins and proanthocyanidins [26], while shorter maceration times result in lower tannin extraction [27].

The aforementioned vinification technologies are primarily used to elevate the concentration of phenols in wine; however, they may also enhance the availability of remaining compounds in the solid parts, for instance in grape pomace constituents. Due to the lack of the available literature with data related to this topic, the aim of this study was to evaluate the impact of pre-fermentative and post-fermentative vinification technologies on bioactive compounds, particularly phenols, and the antioxidant activity of fermented Teran red wine by-products, such as grape skins, seeds, and wine lees. Teran (*Vitis vinifera* L.) is the most widespread red autochthonous variety in Istria [28,29,30], traditionally grown in the north Adriatic area, including the Croatian Istria viticultural subregion [31]. Teran is well known for its deeply colored wines with higher acidity and bold tannins, but with the careful application of adequate canopy management practices and vinification technologies, well-balanced wines with a strong body, high quality, and complex sensory characteristics can be achieved. Based on few researches, different results of total phenolic content (TPC) in Teran grapes and wines can be obtained. In research by [32], the TPC ranges from 2.71 to 3.23 mg/g for berry fresh weight, depending on the viticulture practice, while in wine, the TPC ranges from 821.52 mg/L to 2710.61 mg/L among different vinification treatments [33].

## 2. Materials and Methods

### 2.1. Chemicals and Reagents

Folin & Ciocalteu’s reagent (2N solution), 2,4,6-tris(2-pyrydil)-s-triazine were obtained from Sigma-Aldrich (St. Louis, MO, USA). Ortho-phosphoric acid and hidrochloric acid 37% were supplied from Lach-ner, s. r. o. (Neratovice, Czech Republic). Water, methanol, acetic acid glacial, formic acid, as well as iron (III) chloride hexahydrate, and iron (II) sulphate heptahydrate were purchased from VWR international S.A.S. (Rosny-sous-Bois Cedex, France). Sodium acetate anhydrous and sodium carbonate anhydrous were purchased from Gram-Mol d.o.o. (Zagreb, Croatia), acetonitrile was purchased from Fischer Scientific (Loughborough, UK), gallic acid monohydrate was purchased from Alfa Aesar GmbH & Co KG (Karlsruhe, Germany), and PTFE syringe filters were purchased from FilterBio Membrane Co., Ltd. (Nantong City, China). Water, methanol, acetonitrile, acetic acid glacial, formic, and ortho-phosphoric acid were all HPLC-grade purity. Syringic acid, *trans*-caftaric acid, vanilic acid, caffeic acid, quercetin hydrate, quercetin-3-glucoside, and *trans*-piceid standards were purchased from Sigma-Aldrich (St. Louis, MO, USA). Myricetin and *p*-hydroxybenzoic acid were supplied from Acros Organics (Geel, Belgium). Quercetin-3-glucuronide, kaempferol, procyanidins B1, B2, B3, and C1, (+)-catechin hydrate, (−)-epicatechin, piceatannol, and resveratrol were purchased from Extrasynthese (Genay, France). Anthocyanin standards (delphinidin-3-glucoside chloride, cyanidin-3-glucoside chloride, petunidin-3-glucoside chloride, peonidin-3-glucoside chloride, malvidin-3-glucoside chloride) were purchased from Biosynth Carbosynth (Bratislava, Slovakia). Gallic acid, *p*-coumaric acid, protocatechuic acid, ferulic acid, and taxifolin were obtained from Fluka (Buchs, Switzerland).

### 2.2. Grape Material

The Teran red grapevine variety (*Vitis vinifera* L.) was used for this experiment, which was conducted during the 2020 growing season. The vineyard located in Poreč (Istria wine region, Croatia) was established in 2006 on chromic luvisol (Terra rossa) soil, on a west-facing slope with a 5% inclination. Vines were spaced 2.5 m × 0.8 m, resulting in a planting density of 5000 vines per hectare. The training system was vertically shoot positioned single Guyot, and the vineyard was not irrigated. The vineyard was fertilized annually after harvesting with 800 kg/ha of pelleted organic fertilizer and with 300 kg/ha of PK 20–30 mineral fertilizer. Protective treatments against downy mildew, powdery mildew, and the American grapevine leafhopper (*Scaphoideus titanus*) were applied throughout the growing season, resulting in a total of ten treatments during the season.

At the berry set, approximately two leaves per shoot were manually removed from the fruiting zone in order to obtain a moderately exposed cluster zone. Shoot trimming of vertically positioned shoots was performed at a canopy height of 135 cm at berry set and repeated three weeks later. As Teran is a high-yielding variety requiring cluster thinning to obtain an adequate leaf area/yield ratio [32], 25% of the clusters were removed at the beginning of veraison. The grape yield at harvest was nine tons per hectare. Harvest took place on September 30, with the grapes in healthy, good condition. The sugar content at harvest was 18.9 °Brix, total acidity as tartaric acid was 8.0 g/L, and the pH was 3.20.

The rainfall values and the daily mean temperatures used for the calculation of growing-degree days were obtained by the Croatian Meteorological and Hydrological Service, from a weather station located 200 m from the vineyard. The sum of growing-degree days in the period from 1 April to 30 September in season 2020 was 1917, while the sum of rainfall was 516 mm. The warmest month was August, with mean temperature of 24.6 °C and with the sum of growing-degree days of 453, while the month with the most abundant rainfall was September (210 mm).

### 2.3. Minivinification

In order to obtain wine and winemaking by-products, the application of different pre-fermentative and post-fermentative vinification technologies was performed, as previously reported by [29]. Upon harvest, grapes were processed by destemming and crushing procedures in the experimental wine cellar “Minivinification” owned by Institute of Agriculture and Tourism in Poreč. The grape mash was treated with 5 g/hL of potassium metabisulfite and Aromax. After completing these procedures, an equal amount of homogenized red grape mash was transferred into 220 L stainless steel tanks, where six vinification treatments were conducted, each in three replications (Table 1).

This experiment included a control treatment (K7), with standard 7-day maceration and a maceration/fermentation at a temperature of 24 °C.

Furthermore, three vinification treatments were subjected to pre-fermentative mash cooling at 8 °C (cryomaceration) for 48 h, i.e., 2 days. Two of these treatments were followed by the period considering simultaneous fermentation/maceration at 24 °C and prolonged (extended) post-fermentative maceration in two periods of duration: 13 days and 28 days, with a total duration of 15 days (C15) and 30 days (C30), respectively. In the third treatment after the 2-day pre-fermentative mash cooling and prior to fermentation, the saignée procedure was conducted (CS15). The portion (33%) of the total juice volume was racked, and the concentrated mash then underwent to the period considering simultaneous fermentation/maceration (24 °C) and prolonged post-fermentative maceration in a duration of 13 days, accounting for 15 days in total.

Finally, another two of six vinification treatments were submitted to pre-fermentative mash heating at 50 °C for 48 h, i.e., 2 days (hot pre-fermentative maceration), followed by the period considering simultaneous fermentation/maceration at 24 °C and prolonged post-fermentative maceration in two periods of duration: 13 days and 28 days, with a total duration of 15 days (H15) and 30 days (H30), respectively.

Before distributing the mashes into the tanks, Endozym Rouge (4 g/hL) was added to all mashes. For treatments involving pre-fermentative mash heating, the enzymes were introduced after the heating process. After pre-fermentative procedures, all mashes were inoculated with a selected dry yeast *Saccharomyces cerevisiae* Fermol Mediterranee at 30 g/hL, and Fermol Plus Starter at a dose of 10 g/hL was added to mashes. Fermol Plus H_2_S Free (AEB SPA, Brescia, Italy) was added to mashes on the fourth day of fermentation at a dosage of 10 g/hL. Throughout the maceration period, the cap was manually punched down three times a day using a cap submerging system integrated into the stainless-steel tank. After the end of fermentation, the grape mash was sulphited with potassium metabisulfite and Aromax to achieve a concentration of 20 mg/L of free SO₂. After the maceration ended, the mash was pressed using a pneumatic press (Letina Inox d.o.o., Čakovec, Croatia), which was set to following pressure parameters: 3 cycles at 0.3 bar and 1 cycle at 0.5 bar. Afterwards, the wine was transferred and left to precipitate in stainless steel tanks for 12 days, whereupon they were racked and separated from the lees. All enological products were supplied by AEB SPA, Brescia, Italy.

### 2.4. By-Product Sample Preparation

Grape skin and seeds from by-products were manually separated from the pressed grape pomace using a sieve. Wine lees were collected from the bottom of the tanks after a 12-day long sedimentation followed by racking of the wine and in order to reduce the liquid content, and to obtain compact wine lees; samples prior to lyophilization were centrifuged 3600 rpm for 5 min (Hettich Universal 320 R, Andreas Hettich GmbH & Co. KG, Tuttlingen, Germany). Samples were weighed (30 g of grape seeds, 150 g of grape skins, and 200 g of wine lees), kept at −80 °C (ultra-low temperature freezer Arctiko (ARCTIKO Ltd., Salisbury, UK), and lyophilized using a Labogene ScanVac CoolSafe 55-4 (Allerød, Denmark) freeze dryer, at temperatures from −50 to −55 °C.

During lyophilization, weight loss was monitored by weighing to obtain the percentage of drying and drying yield. Lyophilization was carried out until the mass of the samples was constant. The obtained average percentage of drying for skin samples of all treatments was 76.15 ± 1.20%, meaning that drying yield was 23.85 ± 1.20%, the percentage of drying for grape seed samples was 47.95 ± 1.63%, with a drying yield of 52.05 ± 1.63%, while for wine lees samples, the percentage of drying was 79.37 ± 0.80%, with a drying yield of 20.63 ± 0.80%. After that, samples were vacuum-packed in plastic bags and stored in the dark at 20 °C until extraction was performed.

### 2.5. Extraction

Prior the extraction of bioactive compounds, samples were ground to obtain powdered samples, which were submitted to the extraction procedure [34,35]; the solid–liquid extraction (SLE) method was performed using an extraction solvent composed of acetonitrile–water–formic acid (20:79:1, *v*/*v*/*v*), with a solid to solvent ratio of 1:80 g/mL. The extraction was carried out for one hour in a lab oven at 50 °C, using a magnetic stirrer (RTC basic, IKA, Staufen, Germany) at 500 rpm. After extraction, mixtures of extracts were centrifuged at 4500 rpm for 10 min at 20 °C and filtered using PTFE syringe filters with a pore size of 0.22 μm (FilterBio Membrane Co., Ltd., Nantong City, China). The obtained extracts were used for spectrophotometric and HPLC analysis.

### 2.6. Analysis of Individual Phenolic Compounds

The determination of individual phenolic compounds (anthocyanins, flavan-3-ols, flavonols, phenolic acids, and stilbenes) was conducted by high-performance liquid chromatography (HPLC) using an Agilent Infinity 1260 system (Agilent Technologies, Palo Alto, CA, USA), configured with a DAD and FLD detectors along with a quaternary pump, auto sampler, and column oven. The method that was employed for determination of phenols in fermented grape skins, seeds, and wine lees was developed by [36], with slight modifications. The separation process was carried out on a Zorbax SB-C18 reversed-phase column (250 mm × 4 mm i.d., particle size 5 µm (Agilent Technologies), and thermostated at 50 °C. The solvents used were as follows: water–phosphoric acid (99.5:0.5, *v*/*v*, eluent A) and acetonitrile–water–phosphoric acid (50:49.5:0.5, *v*/*v*/*v*, eluent B), with a flow rate set at 0.9 mL/min. The solvent gradient for eluent B was 0 min, 0%; 2 min, 0%; 7 min, 20%; 35 min 40%; 40 min, 40%; 45 min 80%; 50 min, 100%; 52 min, 100%; 60 min, and 0%; 64 min, 0%. A sample volume of 100 μL was injected onto the column. The chromatograms were captured by the diode array detector (DAD) at a wavelength of 280 nm for hydroxybenzoic acids, 308 nm for stilbenes, 320 nm for hydroxycinnamic acids, 360 nm for flavonol–glycosides and 518 nm for anthocyanins. To detect flavan-3-ols, the fluorescence detector (FLD) was employed, with the excitation set at 225 nm and emission at 320 nm.

Identification was achieved by comparing retention times and/or UV–Vis spectra with the available compound standards. Quantification was performed using calibration equations of the corresponding external standard. The acetyl and *p*-coumaroyl derivates of anthocyanins were determined by matching the relative retention times and spectroscopic parameters (λ max/nm) reported in the paper [36]; quantification was based on a standard equation corresponding to the non-acylated/*p*-coumaroylated anthocyanin compound. In Table S1 in the Supplementary Materials and Data, the parameters of the method such as the retention time, detection wavelengths, calibration equation, coefficient of determination, and compound standard used are reported. The total HPLC phenolic concentration was reported as the sum of all identified phenolic compounds determined by HPLC. In addition, the sum of each compound group was calculated. The results were presented as mg per 100 g of dry weight (DW).

### 2.7. Analysis of Total Phenolic Content

The total phenolic content (TPC) of all samples was determined by the Folin–Ciocalteu colorimetric method according to [37], using a Cary 50 UV/Vis spectrophotometer (Varian Inc., Harbour City, CA, USA). The absorbance was measured against a blank at a wavelength of 765 nm. The standard calibration equation was obtained based on gallic acid with a concentration range from 50 to 1000 mg/L. Results are expressed in mg/100 g of dry matter (mg GAE/100 g DW) as gallic acid equivalents. In Table S2 in the Supplementary Materials and Data, the parameters of the method such as calibration equation and the coefficient of determination are provided.

### 2.8. Analysis of Antioxidant Activity

Antioxidant activity (AA) was determined by employing the ferric reducing/antioxidant power assay (FRAP) according to the method of [38], also using a Cary 50 UV/Vis spectrophotometer (Varian Inc., Harbour City, CA, USA). Aqueous standard solutions of FeSO_4_ × 7H_2_O (0–1000 μmol/L) were used for the calibration equation. The results were expressed as μmol Fe^2+^/100 g sample DW. In Table S2 in the Supplementary Materials and Data, the method parameters including the calibration equation and the coefficient of determination are detailed.

### 2.9. Statistical Data Analysis

The study was performed in three replications, with data analysis based on the averaged values. Statistical analysis was carried out using one-way analysis of variance (ANOVA), and subsequently, Fisher’s least significant difference (LSD) test was applied for mean comparison (*p* ≤ 0.05). In the statistical assessment for the individual phenolic compounds, TPC, and AA of three by-products (grape skins, seeds, and wine lees) obtained after the vinification of Teran red wine, the factor was treatment (control-K7 and five treatments submitted to pre-fermentative and post-fermentative vinification technologies—CS15, C15, H15, C30, and H30). To enhance data interpretation, the dataset was analyzed using principal component analysis (PCA). Statistical assessment was performed using Statistica 10.0. software (Sta-Soft Inc., Tulsa, OK, USA). Furthermore, Pearson’s correlation analysis was employed to examine the relationships between particular values.

## 3. Results and Discussion

As displayed in Table 2, Table 3 and Table 4 in the investigated extracts of fermented Teran grape skins and seeds, as well as wine lees, a total of 25 phenolic compounds were identified and quantified. The obtained results for each wine by-product (grape skins, seeds, and wine lees), are presented in Table 2, Table 3 and Table 4. All the compounds were arranged into groups based on their chemical structure (hydroxybenzoic and hydroxycinnamic acids, stilbenes, flavonols, flavan-3-ols, and anthocyanins), and for a simpler evaluation of the results, the total concentration for each group was provided. Additionally, the total HPLC phenolic concentration was presented as the sum of all identified phenolic compounds determined by HPLC. Although phenolic compounds in grape pomace extracts from various grape varieties have already been studied, many native varieties such as Teran, the most widespread red autochthonous variety in Istria, have not yet been assessed from this aspect.

### 3.1. Individual Phenolic Compounds in Grape Skin Extracts

The concentrations of individual phenolic compounds in Teran fermented grape skin extracts analyzed by HPLC are reported in Table 2.

**Table 2:** Concentration of individual phenolic compounds in grape skin samples obtained after vinification of Teran red wines (mg/100 g DW) detected by HPLC.

Phenolic Compounds	Treatments					
	K7	CS15	C15	H15	C30	H30
**Phenolic acids**						
**Gallic acid**	9.83 ± 0.55 e	14.60 ± 0.60 d	16.80 ± 0.21 c	19.97 ± 0.53 b	17.33 ± 0.61 c	23.15 ± 0.38 a
**Protocatehuic acid**	3.79 ± 0.23 c	4.69 ± 0.50 ab	4.59 ± 0.18 ab	4.35 ± 0.14 b	5.06 ± 0.39 a	3.61 ± 0.12 c
**Vanillic acid**	8.83 ± 0.55 a	0 ± 0 b	0 ± 0 b	0 ± 0 b	0 ± 0 b	0 ± 0 b
**Syringic acid**	10.51 ± 0.25 d	15.46 ± 0.14 c	25.48 ± 0.41 a	20.63 ± 1.07 b	16.20 ± 1.11 c	9.82 ± 0.09 d
**Total detected hydroxybenzoic acids**	**32.95 ± 0.94 f**	**34.76 ± 0.99 e**	**46.86 ± 0.49 a**	**44.94 ± 1.26 b**	**38.59 ± 0.95 c**	**36.58 ± 0.22 d**
**Caftaric acid**	9.45 ± 0.35 d	9.79 ± 0.14 d	14.33 ± 0.21 c	40.05 ± 0.61 a	11.25 ± 0.22 c	29.80 ± 0.43 b
**Total detected hydroxycinnamic acids**	**9.45 ± 0.35 d**	**9.79 ± 0.14 d**	**14.33 ± 0.21 c**	**40.05 ± 0.61 a**	**11.25 ± 0.22 c**	**29.80 ± 0.43 b**
**Stilbenes**						
***trans*** **-piceid**	0 ± 0 e	1.99 ± 0.10 c	1.93 ± 0.09 c	9.08 ± 0.10 a	1.10 ± 0.11 d	8.61 ± 0.07 b
***trans*** **-resveratrol**	0 ± 0 e	4.27 ± 0.46 c	4.43 ± 0.27 c	7.36 ± 0.08 a	3.23 ± 0.15 d	6.17 ± 0.06 b
**Total detected stilbenes**	**0 ± 0 e**	**6.26 ± 0.48 c**	**6.37 ± 0.25 c**	**16.44 ± 0.18 a**	**4.33 ± 0.25 d**	**14.78 ± 0.12 b**
**Flavonols**						
**Quercetin-3-*O*-glucoside**	12.84 ± 0.20 e	17.33 ± 0.12 c	15.10 ± 0.05 d	22.56 ± 0.41 a	9.65 ± 0.08 f	19.13 ± 0.28 b
**Quercetin**	10.85 ± 0.08 e	52.73 ± 2.96 a	24.14 ± 0.95 c	14.40 ± 0.84 d	48.84 ± 0.23 b	48.42 ± 1.69 b
**Kaempferol**	5.37 ± 0.11 e	6.57 ± 0.37 c	7.23 ± 0.11 b	5.87 ± 0.05 d	7.30 ± 0.16 b	8.01 ± 0.28 a
**Total detected flavonols**	**29.06 ± 0.13 e**	**76.63 ± 3.29 a**	**46.47 ± 0.91 c**	**42.83 ± 1.13 d**	**65.79 ± 0.21 b**	**75.56 ± 2.13 a**
**Flavan-3-ols**						
**(+)-Catechin**	18.36 ± 3.27 a	14.35 ± 1.42 b	14.85 ± 1.51 ab	14.66 ± 1.02 b	16.87 ± 1.38 ab	13.65 ± 2.74 b
**(−)-Epicatechin**	2.79 ± 0.09 c	6.67 ± 0.56 b	6.61 ± 0.33 b	6.93 ± 0.29 ab	8.14 ± 0.42 a	7.55 ± 0.39 a
**Procyanidin B1**	10.35 ± 0.69 d	13.60 ± 0.42 ab	12.44 ± 0.32 c	12.56 ± 0.67 bc	14.90 ± 0.29 a	14.56 ± 0.99 a
**Procyanidin B2**	2.61 ± 0.15 d	7.61 ± 0.42 b	6.85 ± 0.07 c	6.54 ± 0.40 c	9.08 ± 0.14 a	8.58 ± 0.4 a
**Procyanidin B3**	3.30 ± 0.11 c	4.68 ± 0.17 b	4.50 ± 0.09 b	4.33 ± 0.41 b	5.20 ± 0.28 a	5.59 ± 0.17 a
**Procyanidin C1**	6.30 ± 1.19 a	1.73 ± 0.02 b	1.80 ± 0.10 b	1.88 ± 0.13 b	1.80 ± 0.22 b	2.44 ± 0.07 b
**Total detected flavan-3-ols**	**43.71 ± 5.25 c**	**48.63 ± 2.67 bc**	**47.05 ± 2.20 bc**	**46.90 ± 2.87 bc**	**55.99 ± 2.12 a**	**52.37 ± 3.24 ab**
**Anthocyanins**						
**Delphinidin-3-*O*-glucoside**	12.31 ± 0.15 f	45.91 ± 0.71 a	29.58 ± 0.58 c	11.54 ± 0.23 e	31.34 ± 0.07 b	18.60 ± 0.42 d
**Cyanidin-3-*O*-glucoside**	1.23 ± 0.04 d	2.86 ± 0.08 b	2.55 ± 0.02 a	0.74 ± 0.02 e	1.60 ± 0.06 c	0.83 ± 0.03 f
**Petunidin-3-*O*-glucoside**	17.67 ± 0.03 d	43.46 ± 0.16 a	34.48 ± 0.30 b	15.76 ± 0.23 f	32.26 ± 0.28 c	19.87 ± 0.40 e
**Peonidin-3-*O*-glucoside**	14.40 ± 0.40 d	27.71 ± 0.33 a	20.34 ± 0.31 b	11.34 ± 0.20 e	17.15 ± 0.34 c	11.19 ± 0.20 e
**Malvidin-3-*O*-glucoside**	206.93 ± 5.97 d	415.33 ± 3.94 a	352.97 ± 2.77 b	183.87 ± 3.47 f	342.12 ± 4.16 c	213.87 ± 3.42 d
**Peonidin-3-*O*-acetilglucoside**	3.27 ± 0.25 e	9.95 ± 0.35 a	5.77 ± 0.10 c	3.01 ± 0.18 e	7.93 ± 0.95 b	4.86 ± 0.08 d
**Malvidin-3-*O*-acetilglucoside**	40.08 ± 0.64 c	76.80 ± 2.02 a	61.60 ± 1.03 b	34.96 ± 0.57 d	63.64 ± 3.40 b	40.49 ± 0.66 c
**Peonidin-3-*O*-coumarylglucoside**	13.04 ± 0.2 b	16.97 ± 0.19 a	13.77 ± 0.46 b	9.46 ± 0.25 c	12.89 ± 1.11 b	10.30 ± 0.31 c
**Malvidin-3-*O*-coumarylglucoside**	109.70 ± 1.69 c	163.48 ± 3.35 a	142.23 ± 1.43 b	92.44 ± 4.88 e	143.92 ± 0.83 b	102.73 ± 3.43 d
**Total detected anthocyanins**	**418.63 ± 8.27 c**	**802.47 ± 10.24 a**	**663.29 ± 5.95 b**	**363.11 ± 8.91 d**	**652.86 ± 9.07 b**	**422.74 ± 8.84 c**
**Total detected phenolic compounds**	**533.80 ± 3.57 e**	**978.54 ± 14.96 a**	**824.37 ± 4.43 b**	**554.27 ± 13.67 d**	**828.81 ± 9.96 b**	**631.82 ± 14.28 c**

Each value represents the mean ± standard deviation, with n = 3. Significant differences at the *p* ≤ 0.05 level (LSD test) are indicated with lowercase letters. Treatments: control—standard 7-day maceration (K7); pre-fermentative cryomaceration (8 °C, 48 h) followed by a 13-day (C15) and 28-day (C30) period of simultaneous fermentation/maceration and extended post-fermentative maceration; pre-fermentative cryomaceration (8 °C, 48 h) followed by saignée procedure, and 13-day period of simultaneous fermentation/maceration and extended post-fermentative maceration (CS15); pre-fermentative hot maceration at (50 °C, 48-h) followed by a 13-day (H15) and 28-day (H30) period of simultaneous fermentation/maceration and extended post-fermentative maceration.

The total concentration of phenols detected by HPLC ranged from 533.80 mg/100 g of dry weight (DW) in the control treatment (K7) to 978.54 mg/100 g DW in treatment with pre-fermentative cooling, the saignée procedure, and 15-day maceration (CS15). The saignée procedure, which involves the removal of a portion of the must prior to the onset of fermentation, effectively increases the solid (i.e., skins plus seeds)-to-pulp ratio [39]. The total concentration of phenolic compounds in grape skin samples in all the treatments was significantly higher compared to the control (K7), as noted in a previous paper [29] which reported the phenolic profile of wines from which these by-products originated. The treatments where pre-fermentative hot maceration was applied (H15 and H30) showed the most elevated concentrations in the corresponding wines [29] as a result of a more intense extraction of phenolic compounds from the skins into the liquid phase supported by heat. Consequently, such an exhaustion of pomace led to a lower concentration of phenolic compounds in grape skin samples of these treatments in this study (H15 and H30), when compared to the other three treatments, that is, CS15, C15, and C30 (Table 2).

**Figure 1:**
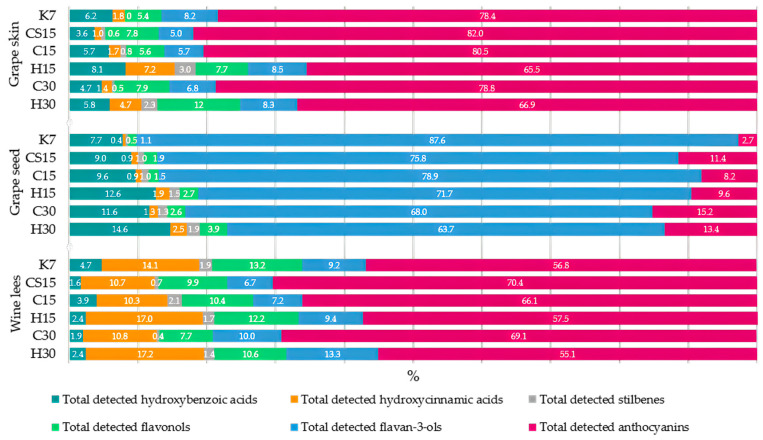
Percentage (%) of each group of phenolic compounds in total phenol concentration determined by HPLC analysis in samples of particular by-product (grape skins, seeds, and wine lees) obtained after vinification of Teran red wines. Treatments: control—standard 7-day maceration (K7); pre-fermentative cryomaceration (8 °C, 48 h) followed by a 13-day (C15) and 28-day (C30) period of simultaneous fermentation/maceration and extended post-fermentative maceration; pre-fermentative cryomaceration (8 °C, 48 h) followed by saignée procedure, and 13-day period of simultaneous fermentation/maceration and extended post-fermentative maceration (CS15); pre-fermentative hot maceration at (50 °C, 48-h) followed by a 13-day (H15) and 28-day (H30) period of simultaneous fermentation/maceration and extended post-fermentative maceration.

The aforementioned CS15 treatment had the highest concentration of total phenolic compounds in grape skin samples, followed by the other two treatments submitted to pre-fermentative cooling (C15 and C30). Although both applied pre-fermentative procedures, i.e., mash cooling and heating to facilitate the extraction of phenols from a solid phase into a liquid phase [24,40], in this investigation with Teran grape skin samples, it was evident that heating had a more profound impact on the release of these compounds compared to cooling. Consequently, a higher concentration of phenols remained in grape skin samples from the treatments with pre-fermentative cooling. The highest concentration of total phenolic compounds found in CS15 grape skins suggests that the lower liquid-to-solid ratio obtained in this system after the removal of a significant portion of grape juice (33%) prior to fermentation reduced the initial concentration gradient that drives the diffusion of phenols from grape solids to juice. The saturation of grape juice with phenolics from the skins led to equilibrium concentrations being reached more quickly, causing the diffusion to stop or at least significantly slow down, leaving higher amounts in the skins. Among the analyzed phenolic compounds in Teran grape skins, the most quantitatively abundant were anthocyanins, ranging from 65.5% in the H15 treatment to 82.0% in the CS15 treatment of the total detected phenolic compounds (HPLC), with malvidin-3-*O*-glucoside, malvidin-3-*O*-acetilglucoside, and malvidin-3-*O*-coumarylglucoside as the most representative compounds (Figure 1).

This suggests that anthocyanins are the most easily extractable phenolic compounds during vinification compared to other groups of phenolic compounds [41]. Indeed, skins, in which anthocyanins are mostly located, are more altered than seeds by the procedures such as pressing, crushing, and maceration [12]. The total concentration of detected anthocyanins in grape skin samples ranged from 363.11 in the H15 treatment to 802.47 mg/100 g DW in the CS15 treatment. When compared to other studies, in the work by [42], the total anthocyanin values detected by HPLC in berry skin samples after vinification ranged from 289.46 to 934.67 mg/100 g DW, depending on the variety, which fall within the range of values in our investigation. The highest content of anthocyanins in Teran grape skins was detected in treatments subjected to pre-fermentative cryomaceration (CS15, C15, and C30), while in treatments submitted to pre-fermentative heating, grape skins were probably more exhausted, exposing lower concentrations of anthocyanins. In this sense, it can be assumed that higher temperatures promote greater anthocyanin extraction into the liquid phase, i.e., into wine compared to lower temperatures during cold maceration due to heat supported breakdown of grape cell walls and softening of the skins. However, the differences between anthocyanin concentrations in the corresponding wines in our previous report were of a lower extent [29] compared to those obtained for grape skins in this study. It must be kept in mind that during maceration supported by heating, anthocyanins also undergo thermal degradation and increased polymeric pigment formation by binding with tannins, which could have also affected their amounts in both wine and grape skins. As well, anthocyanins in wine are altered by other phenomena during maceration, such as adsorption onto skins and larger molecules, as well as yeasts, followed by precipitation.

Concerning the total concentrations of detected flavonols in fermented grape skins, concentrations found in all treatments were higher in comparison to the control (K7). Significantly, the highest concentration was found in treatments submitted to the saignée procedure (CS15), and in the H30 treatment where pre-fermentative heating, followed by a longer maceration duration, was performed. Unlike for anthocyanins, for which pre-fermentative temperature seemed to have a decisive impact, flavonols were more affected by skin contact duration: the treatments that included a longer maceration period, C30 and H30, contained higher total flavanol concentrations than their 15-day counterparts, C15 and H15. Flavonols are vacuolar substances co-located with anthocyanins; however, their extraction follows a slower pattern compared to anthocyanins, primarily due to differences in polarity [40,43], which was possibly among the main factors that affected their dynamic distribution between the solid and liquid phase during the treatment, depending on the treatment type. Other phenomena caused by heat, such as increased solubility, thermal degradation, oxidative changes, and polymerization could have also had an effect.

Among phenolic acids, hydroxybenzoic (HB) acids were represented in higher quantity in fermented grape skins than hydroxycinammic acids (HC), with syringic and gallic acid as the most abundant. The absence of other phenolic acids, especially HC acids, including hydroxycinnamoyltartrates such as coutaric and fertaric acid, along with free HC acids, like caffeic, *p*-coumaric and ferulic acid, suggest a possibility of their complete dissolution in the liquid phase in wine. Both treatments that were subjected to a 15-day prolonged maceration (C15 and H15), regardless of the pre-fermentative mash procedure (cooling or heating), retained more HB compounds within the grape skin structure. This indicates that during the 30-day prolonged maceration, the extraction of HB acids into wine was stronger, which is evident when observing the HB content of corresponding Teran wines [29], possibly partly caused by the hydrolysis of gallotannins and condensed tannins, such as esters of gallic acid with flavan-3-ols [44,45]. When observing the particular pre-fermentative procedure, i.e., cooling or heating, slightly but significantly higher concentrations of total HB acids were found in treatments that involved cooling, C15 or C30, in comparison to those that included heating in the pre-fermentative phase, such as H15 or H30, respectively. Conversely to HB acids, when observing the only detected HC acid, caftaric acid, the effect of pre-fermentative heating was more pronounced on retaining the HC acids, with the highest concentrations found in H15 and H30, despite the maceration duration. It is possible that heating reduced the activity of cinnamoyl esterases, which are responsible for the hydrolysis of hydroxycinnamoyltartrates and the release of free forms.

Concerning the group of stilbenes, *trans*-piceid and its aglycon *trans*-resveratrol were detected in skins of all treatments except the control treatment (K7). Grape skin is a particularly good source of resveratrol [46], with values in Teran skin samples ranging from 3.23 to 7.26 mg/100 g DW, which is in accordance with other investigations on fermented grape skins by [46]. Onwards, the highest content was determined in the H15 treatment with pre-fermentative heating and 15-day maceration (H15), with a value of 16.44 mg/100 g DW, followed by another heating treatment, H30 (30-day prolonged maceration), with a value of 14.78 mg/100 g DW. In control treatment (K7) the stilbenes even were not detected. When compared to other research, grape skin samples from the fermented pomace of the Prokupac red variety showed a total of 5.77 mg/100 g DW for stilbenoids [47]. Results from our study were either similar or in some cases even 2.9-fold higher than those detected in previously mentioned works. The obtained results suggest that pre-fermentative hot maceration might have had a decisive influence on the availability of stilbene compounds in Teran grape skin structures. Another finding contributing to this fact is that a previous study on the phenolic concentration in Teran wine [29], wherefrom these by-products originated, also revealed that the highest stilbene content was observed in wine samples obtained from H15 and H30 treatments, which corroborates this hypothesis. When comparing 15 and 30 days of maceration, within the particular pre-fermentative procedure, i.e., cooling or heating, fermented grape skins from the treatments with a shorter maceration duration of 15 days, C15 or H15, had slightly but significantly higher stilbene concentrations than those with longer skin contact, C30 or H30, respectively. CS15 grape skins contained stilbene concentrations comparable to those found in the C15 treatment. Flavan-3-ol concentrations in fermented grape skins were also significantly affected by the investigated treatments. Regarding the total detected flavan-3-ols, concentrations found in both treatments, with pre-fermentative mash cooling and mash heating and 30-day extended maceration (C30 and H30), were significantly higher in comparison to the control treatment (K7). Considering individual flavan-3-ols, in most cases, the significantly highest concentrations (procyanidin B1, procyanidin B2, procyanidin B3, (-)-epicatechin) were found in C30 and H30 treatments, while the control treatment K7 was characterized by the lowest levels. It is evident that a more highlighted effect on the concentration of flavan-3-ols had a longer, 30-day maceration duration, regardless of the pre-fermentative mash procedure, i.e., cooling and heating (C30 and H30), meaning that the maceration length was the main affecting factor. A similar relationship between the concentrations of flavan-3-ols across the treatments was found in our previous study for the corresponding wines from the same experiment, where C30 and H30 wines contained the highest concentrations [29]. The fact that C30 and H30 skins were not deprived of flavan-3-ols, which could be expected, means that the determined differences cannot be explained simply by the longer time available for the diffusion of flavan-3-ols from skins into wine. One of the possible causes could be the prolonged contact of grape skins with acidic fermenting must or wine in C30 and H30 treatments, which allowed for a longer time for the acid hydrolysis of condensed tannins and the release of monomeric and oligomeric flavan-3-ols to happen, both in wine and within skin structures. It must be also kept in mind that the concentrations of phenolic compounds in wine, especially that of flavan-3-ols, were certainly affected by their extraction from seeds, which is known to be facilitated and accelerated in the presence of ethanol during post-fermentative maceration. The complexity of the possible interactive effects becomes more obvious, considering that the pre-fermentation temperature had a significant impact on the concentrations of flavan-3-ols in wine, as reported previously [29]. (+)-Catechin and especially procyanidin C1 behaved differently, with the highest concentration found in the control K7 treatment grape skins, possibly due to their different solubility and availability in tannin complexes in comparison to other flavan-3-ols, among other reasons.

### 3.2. Individual Phenolic Compounds in Grape Seed Extracts

The concentration of individual phenolic compounds in grape seed extracts obtained in the vinification of Teran red wine determined by HPLC are reported in Table 3.

**Table 3:** Concentration of individual phenolic compounds in grape seed samples obtained after vinification of Teran red wines (mg/100 g DW) detected by HPLC.

Phenolic Compounds	Treatments					
	K7	CS15	C15	H15	C30	H30
**Phenolic acids**						
**Gallic acid**	26.00 ± 0.90 a	24.50 ± 2.13 a	25.68 ± 1.38 a	24.14 ± 0.91 a	24.98 ± 0.81 a	24.17 ± 0.94 a
**Protocatehuic acid**	2.41 ± 0.04 ab	2.21 ± 0.27 b	2.64 ± 0.01 a	2.52 ± 0.04 a	2.14 ± 0.06 b	1.89 ± 0.07 c
**Syringic acid**	4.68 ± 0.77 a	3.29 ± 0.08 b	3.32 ± 0.34 b	2.96 ± 0.53 c	2.31 ± 0.31 d	1.87 ± 0.13 e
**Total detected hydroxybenzoic acids**	**33.09 ± 1.54 a**	**30.01 ± 2.30 bc**	**31.64 ± 1.49 ab**	**29.62 ± 0.81 bc**	**29.43 ± 1.09 c**	**27.93 ± 1.14 c**
**Caftaric acid**	1.63 ± 0.71 e	2.89 ± 0.04 d	2.87 ± 1.02 d	4.49 ± 0.76 b	3.19 ± 0.75 c	4.81 ± 0.23 a
**Total detected hydroxycinnamic acids**	**1.63 ± 0.71 e**	**2.89 ± 0.04 d**	**2.87 ± 1.02 d**	**4.49 ± 0.76 b**	**3.19 ± 0.75 c**	**4.81 ± 0.23 a**
**Stilbenes**						
***trans*** **-piceid**	1.06 ± 0.04 c	1.13 ± 0.07 bc	1.05 ± 0.25 c	1.53 ± 0.38 a	0.91 ± 0.26 d	1.20 ± 0.04 b
***trans*** **-resveratrol**	1.19 ± 0.51 d	2.05 ± 0.15 b	2.11 ± 0.11 b	2.10 ± 0.11 b	2.40 ± 0.14 a	2.35 ± 0.02 a
**Total detected stilbenes**	**2.25 ± 0.55 d**	**3.17 ± 0.20 b**	**3.16 ± 0.16 b**	**3.63 ± 0.28 a**	**3.31 ± 0.11 b**	**3.55 ± 0.05 a**
**Flavonols**						
**Quercetin-3-*O*-glucoside**	0.98 ± 0.37 e	1.66 ± 0.26 c	1.20 ± 0.56 d	2.14 ± 0.69 a	1.00 ± 0.39 e	1.75 ± 0.03 b
**Quercetin**	3.43 ± 0.10 c	3.86 ± 0.6 b	3.06 ± 0.04 c	3.39 ± 0.51 c	4.68 ± 0.24 a	4.87 ± 0.06 a
**Kaempferol**	0.44 ± 0.23 c	0.86 ± 0.14 a	0.70 ± 0.04 b	0.86 ± 0.08 a	0.81 ± 0.03 a	0.86 ± 0.01 a
**Total detected flavonols**	**4.85 ± 0.70 c**	**6.38 ± 0.99 b**	**4.96 ± 0.63 c**	**6.38 ± 0.27 b**	**6.49 ± 0.19 b**	**7.47 ± 0.04 a**
**Flavan-3-ols**						
**(+)-Catechin**	142.18 ± 30.20 a	92.51 ± 2.77 b	93.94 ± 25.56 b	57.36 ± 3.01 c	62.91 ± 16.12 c	43.72 ± 0.33 d
**(−)-Epicatechin**	99.65 ± 24.42 a	59.00 ± 1.36 b	62.61 ± 18.48 b	34.72 ± 1.17 d	39.28 ± 10.04 c	25.28 ± 0.26 e
**Procyanidin B1**	42.11 ± 5.57 a	31.14 ± 0.81 b	30.91 ± 3.70 b	24.69 ± 2.09 c	21.86 ± 3.09 d	17.43 ± 0.64 e
**Procyanidin B2**	48.17 ± 5.26 a	37.07 ± 2.27 b	38.92 ± 5.53 b	29.19 ± 1.67 c	26.74 ± 4.32 d	19.99 ± 0.57 e
**Procyanidin B3**	37.25 ± 6.11 a	25.83 ± 0.8 b	26.61 ± 6.41 b	17.23 ± 1.62 c	15.74 ± 2.61 c	11.65 ± 0.29 d
**Procyanidin C1**	9.53 ± 1.30 a	7.10 ± 0.21 c	7.57 ± 1.39 b	5.49 ± 0.35 d	5.34 ± 1.10 d	3.86 ± 0.11 e
**Total detected flavan-3-ols**	**378.89 ± 72.53 a**	**252.65 ± 3.73 b**	**260.55 ± 60.87 b**	**168.67 ± 7.59 c**	**171.88 ± 37.22 c**	**121.93 ± 1.02 d**
**Anthocyanins**						
**Delphinidin-3-*O*-glucoside**	2.20 ± 0.84 e	3.65 ± 0.35 a	3.04 ± 0.15 c	2.83 ± 0.19 d	3.30 ± 0.35 b	2.82 ± 0.14 d
**Cyanidin-3-*O*-glucoside**	0.11 ± 0.06 c	0.21 ± 0.03 a	0.16 ± 0.03 b	0.12 ± 0.02 c	0.15 ± 0.03 b	0.10 ± 0 d
**Petunidin-3-*O*-glucoside**	1.19 ± 0.74 d	2.50 ± 0.34 a	1.94 ± 0.08 bc	1.77 ± 0.02 c	2.20 ± 0.35 b	1.86 ± 0.02 c
**Peonidin-3-*O*-glucoside**	0.45 ± 0.62 d	1.51 ± 0.31 a	0.97 ± 0.03 c	0.91 ± 0.13 c	1.20 ± 0.18 b	0.96 ± 0.05 c
**Malvidin-3-*O*-glucoside**	5.11 ± 8.61 d	20.05 ± 3.68 a	14.00 ± 1.41 b	11.47 ± 4.61 c	20.87 ± 4.76 a	13.81 ± 0.09 b
**Malvidin-3-*O*-acetilglucoside**	1.15 ± 2.01 d	4.11 ± 0.55 a	2.96 ± 0.44 b	2.20 ± 1.57 c	4.50 ± 1.00 a	2.68 ± 0.04 b
**Malvidin-3-*O*-coumarylglucoside**	1.51 ± 2.67 d	6.11 ± 1.27 a	3.93 ± 0.41 b	3.22 ± 1.38 c	6.22 ± 1.82 a	3.49 ± 0.08 c
**Total detected anthocyanins**	**11.72 ± 15.55 d**	**38.15 ± 6.51 a**	**27.02 ± 2.54 b**	**22.50 ± 7.91 c**	**38.44 ± 8.50 a**	**25.72 ± 0.42 b**
**Total detected phenolic compounds**	**432.42 ± 56.34 a**	**333.25 ± 5.75 b**	**330.19 ± 62.18 b**	**235.3 ± 1.79 d**	**252.73 ± 45.5 c**	**191.41 ± 2.37 e**

Each value represents the mean ± standard deviation, with n = 3. Significant differences at the *p* ≤ 0.05 level (LSD test) are indicated with lowercase letters. Treatments: control—standard 7-day maceration (K7); pre-fermentative cryomaceration (8 °C, 48 h) followed by a 13-day (C15) and 28-day (C30) period of simultaneous fermentation/maceration and extended post-fermentative maceration; pre-fermentative cryomaceration (8 °C, 48 h) followed by saignée procedure, and 13-day period of simultaneous fermentation/maceration and extended post-fermentative maceration (CS15); pre-fermentative hot maceration at (50 °C, 48-h) followed by a 13-day (H15) and 28-day (H30) period of simultaneous fermentation/maceration and extended post-fermentative maceration.

Considering the sum of all the detected phenolic compounds in Teran grape seed samples, values ranged up to 432.42 mg/100 g DW in the control treatment extract (K7). The majority of the seed phenolic composition was represented by flavan-3-ols, which made 87.6% of the total phenols detected in grape seeds, with (+)-catehin as the most represented compound (Figure 1, Table 3). When observing the total detected flavan-3-ols in grape seeds, the most elevated concentration was found in the control treatment (K7), with a concentration of 378.89 mg/100 g DW, and the lowest was found in H30 treatment with a value of 121.93 mg/100 g DW, where the latter corresponds to results in investigation [47], which found sum values of flavan-3-ols and procyanidins of 141.4 mg/100 g DW. In line with this observation, it is evident that the applied pre-fermentative and post-fermentative vinification technologies in other treatments (CS15, C15, H15, C30, and H30) strongly affected the extraction of all detected phenols, as well as flavan-3-ols, from seed to wine. This effect was already noticeable in Teran wines obtained from the same vinification treatments in our previous study [29], where the lowest amount of flavan-3-ols and total detected phenolic compounds was found in wine from the control treatment (K7), meaning the lowest quantity of phenols was extracted from grape seeds to wine during the standard 7-day maceration. In reports published by [41,48], it was also reported that the phenolic content in the case of grape seeds consists almost exclusively of flavan-3-ols such as catechin. It is well known that the extraction dynamics of flavan-3-ol and proanthocyanidins from seeds during mash maceration becomes more intense during prolonged macerations. This was also highlighted in [49], who reported that winemaking practices such as extended maceration are particularly effective in enhancing tannin extraction from seeds. Extended maceration is one of the most commonly used methods when the objective is to increase the tannin concentration in wine [39]. Indeed, as the alcohol content rises during the winemaking process, tissues become more permeable, allowing low molecular weight tannins to be released from seeds into wine around the mid-point of fermentation [12]. Due to stated facts, when considering the impact of applied vinification procedures on Teran grape seed phenolic composition, an extended 30-day maceration, with treatments subjected to particular pre-fermentative mash procedure, cryomaceration, or hot maceration (C30 or H30), expressed high influence on the diffusion of flavan-3-ols into the liquid, in comparison to 15-day maceration treatments (C15 or H15). Furthermore, when comparing pre-fermentative procedures, cryomaceration, or hot maceration, with a particular maceration duration, i.e., 15 or 30 days, pre-fermentative cryomaceration (CS15, C15 or C30) demonstrated a stronger effect on retaining higher concentrations of all the detected compounds within the seeds, suggesting that this technology more effectively preserves phenolic compounds in grape seed tissues, in relation to pre-fermentative hot maceration. The impact of hot maceration on the extraction of phenols was also evident in the case of the H15 treatment, whose seeds retained similar quantities of phenols and flavan-3-ols to those found in C30 seeds, but significantly lower than those observed in the C15 treatment. The saignée treatment (CS15) exhibited significantly equal concentrations of total detected phenols to those found in C15 seeds. The second predominant group of phenolic compounds in Teran seed samples, following the most representative flavan-3-ols, were hydroxybenzoic acids (HB), ranging from 7.7% in K7 treatment, respectively, to 14.6% in the H30 treatment, when compared with the total detected phenolic compounds by HPLC in grape seed samples, with gallic acid as the most abundant compound (Figure 1, Table 3). Similar findings were reported by [47], who also found flavan-3-ols and hydroxybenzoic acids to be the most dominant grape seed phenols. HB acid concentrations in Teran grape seed samples were under the influence of an extended post-fermentative maceration of 30 days, which possibly improved solvent efficiency, resulting in the lower quantities determined in seed samples of those treatments (C30 and H30). A similar pattern of HB acid extraction among treatments was reported for grape skin samples. The effect of extended post-fermentative maceration on the release of HB acids from both grape skins and seeds was clearly evident in wines originating from the same treatments, exhibiting the highest values in the C30 and H30 treatments [29]. Regarding the detected stilbenes in Teran grape seed extracts, values ranged from 2.25 to 3.63 mg/100 g DW, with the control treatment (K7) having the lowest concentration (Table 3). The obtained values for *trans*-piceid and *trans*-resveratrol were in agreement with the findings of a group of authors investigating grape seed extracts obtained from different grape varieties after vinification [42]. They also noted that the transfer of resveratrol from grape seeds to the wine is inhibited due to its polar characteristics. In our investigation concerning Teran grape seed extracts, stilbenes significantly exhibited the most elevated concentration in treatments with pre-fermentative hot maceration, regardless of the maceration duration (H15 and H30), as well as in grape skin samples (Table 3). It is possible that hot maceration can disrupt bonds between bounded compounds, releasing free stilbene compounds into the must; however, heating might also preserve the stability of these valuable bioactive compounds, which is the reason for the highest concentrations of stilbenes in the H15 and H30 treatments in both by-products (skin and seeds) and in Teran wines [29].The presence of anthocyanins in grape seeds is not common, but traces usually remain when the seeds are separated from the skins [50] due to the fixation of these compounds onto solid grape parts [51]. Concerning this fact, a slight value of anthocyanins in Teran grape seed extracts were detected, with the highest concentration found in the treatment in which the saignée procedure was employed (CS15) (Table 3). The saignée treatment (CS15) was also highlighted as the one that enhanced the accumulation of anthocyanins in grape skins due to a greater solid-to-liquid ratio (Table 2).

Concerning hydroxycinnamic (HC) acids and flavonols, they were presented in low quantities in grape seed extracts, both significantly showing the most elevated content in the H30 treatment. Higher quantities might have been caused by the re-adsorption of these compounds on the outer part of the seed structure during the longer maceration duration, as was the case for anthocyanins [51].

### 3.3. Individual Phenolic Compounds in Wine Lees Extracts

Wine lees are a sludge material made of intact or partially degraded yeast cells and other insoluble particles [52] that accumulate at the bottom of wine tanks after the alcoholic fermentation. Their composition depends on numerous parameters mainly related to the types of yeast and grapes used and to the vinification method, resulting in a wide compositional heterogeneity [53]. Wine lees are rich in organic matter and polyphenols, making them valuable in food, cosmetics, and pharmaceuticals due to their antioxidant, antimicrobial, anti-inflammatory, and cardioprotective properties [53,54,55]. Due to the notable content of minerals, wine lees are useful in recovering salts and are used as fertilizers [10,54,56]. The concentration of individual phenolic compounds in Teran wine lees samples analyzed by HPLC is reported in Table 4.

**Table 4:** Concentration of individual phenolic compounds in wine lees samples obtained after vinification of Teran red wines (mg/100 g DW) detected by HPLC.

Phenolic Compounds	Treatments					
	K7	CS15	C15	H15	C30	H30
**Phenolic acids**						
**Gallic acid**	3.93 ± 0.25 e	7.70 ± 0.13 c	6.96 ± 0.32 d	11.15 ± 0.43 b	11.08 ± 0.44 b	14.06 ± 0.56 a
**Syringic acid**	5.89 ± 0.16 b	0 ± 0 c	7.22 ± 0.30 a	0 ± 0 c	0 ± 0 c	0 ± 0 c
**Vanillic acid**	5.00 ± 0.61 b	0 ± 0 c	5.52 ± 0.31 a	0 ± 0 c	0 ± 0 c	0 ± 0 c
**Total detected hydroxybenzoic acids**	**14.82 ± 0.98 b**	**7.7** **0** **± 0.13 d**	**19.70 ± 0.97 a**	**11.15 ± 0.43 c**	**11.08 ± 0.44 c**	**14.06 ± 0.56 b**
**Caftaric acid**	44.52 ± 3.70 e	53.00 ± 0.48 d	52.22 ± 0.48 d	80.12 ± 0.49 b	62.97 ± 1.49 c	102.47 ± 1.99 a
**Total detected hydroxycinnamic acids**	**44.52 ± 3.70 e**	**53.00 ± 0.48 d**	**52.22 ± 0.48 d**	**80.12 ± 0.49 b**	**62.97 ± 1.49 c**	**102.47 ± 1.99 a**
**Stilbenes**						
***trans*** **-piceid**	2.61 ± 0.10 f	3.67 ± 0.04 c	4.15 ± 0.05 d	7.81 ± 0.38 b	2.49 ± 0.06 e	8.56 ± 0.22 a
***trans*** **-resveratrol**	3.46 ± 0.21 b	0 ± 0 c	6.29 ± 0.48 a	0 ± 0 c	0 ± 0 c	0 ± 0 c
**Total detected stilbenes**	**6.07 ± 0.30 d**	**3.67 ± 0.04 e**	**10.44 ± 0.45 a**	**7.81 ± 0.38 c**	**2.49 ± 0.06 f**	**8.56 ± 0.22 b**
**Flavanols**						
**Quercetin-3-*O*-glucoside**	3.93 ± 0.65 d	7.48 ± 0.10 c	6.85 ± 0.14 c	15.66 ± 1.61 b	4.37 ± 0.05 d	19.68 ± 0.49 a
**Myricetin**	5.58 ± 0.51 c	7.31 ± 0.87 b	10.66 ± 1.04 a	10.33 ± 0.87 a	7.94 ± 0.21 b	9.80 ± 0.26 a
**Quercetin**	28.76 ± 1.59 a	29.71 ± 2.47 a	31.52 ± 2.51 a	27.59 ± 2.44 a	28.44 ± 0.22 a	28.73 ± 4.46 a
**Kaempferol**	3.49 ± 0.12 e	4.37 ± 0.14 b	3.68 ± 0.19 de	3.85 ± 0.02 cd	4.07 ± 0.29 bc	5.10 ± 0.27 a
**Total detected flavonols**	**41.75 ± 2.88 e**	**48.88 ± 3.5 cd**	**52.71 ± 3.82 bc**	**57.43 ± 4.48 ab**	**44.82 ± 0.34 de**	**63.3 ± 5.36 a**
**Flavan-3-ols**						
**(+)-Catechin**	17.89 ± 0.75 c	15.31 ± 0.45 d	15.74 ± 0.55 d	19.84 ± 0.23 b	20.05 ± 0.67 b	29.46 ± 0.54 a
**(−)-Epicatechin**	1.53 ± 0.47 d	3.48 ± 0.13 c	4.44 ± 0.24 c	4.33 ± 2.54 c	8.48 ± 0.33 b	12.02 ± 1.27 a
**Procyanidin B1**	4.40 ± 0.52 e	6.41 ± 0.38 d	6.99 ± 0.86 d	8.83 ± 0.06 c	13.06 ± 0.31 b	17.83 ± 0.68 a
**Procyanidin B2**	2.22 ± 0.35 f	3.54 ± 0.02 e	4.37 ± 0.21 d	5.01 ± 0.08 c	9.78 ± 0.22 b	11.69 ± 0.19 a
**Procyanidin B3**	2.32 ± 0.29 d	3.39 ± 0.28 c	3.59 ± 0.07 c	3.69 ± 0.03 c	4.53 ± 0.12 b	6.16 ± 0.27 a
**Procyanidin C1**	0.60 ± 0.05 e	1.05 ± 0.04 d	1.21 ± 0.11 c	2.56 ± 2.16 ab	2.36 ± 0.06 ab	2.16 ± 0.05 b
**Total detected flavan-3-ols**	**28.95 ± 2.41 e**	**33.18 ± 1.2 de**	**36.34 ± 1.72 d**	**44.26 ± 3.80 c**	**58.25 ± 1.69 b**	**79.33 ± 2.78 a**
**Anthocyanins**						
**Delphinidin-3-*O*-glucoside**	5.26 ± 0.35 d	14.61 ± 0.33 a	14.65 ± 0.48 a	12.68 ± 0.32 c	13.6 ± 0.6 b	13.31 ± 0.31 bc
**Cyanidin-3-*O*-glucoside**	0.33 ± 0.02 e	1.09 ± 0.01 a	0.79 ± 0.06 b	0.56 ± 0.03 d	0.11 ± 0.01 f	0.71 ± 0.03 c
**Petunidin-3-*O*-glucoside**	6.68 ± 0.26 e	16.65 ± 0.35 b	15.29 ± 0.16 c	12.78 ± 0.17 d	17.35 ± 0.52 a	15.63 ± 0.31 c
**Peonidin-3-*O*-glucoside**	4.30 ± 0.10 e	10.84 ± 0.18 a	8.59 ± 0.05 c	7.36 ± 0.17 d	10.16 ± 0.22 b	8.75 ± 0.17 c
**Malvidin-3-*O*-glucoside**	89.00 ± 1.73 e	179.35 ± 4.05 b	168.78 ± 0.98 c	137.23 ± 0.80 d	207.12 ± 3.82 a	171.41 ± 2.95 c
**Peonidin-3-*O*-acetilglucoside**	2.14 ± 0.03 c	3.87 ± 0.32 b	4.53 ± 0.16 a	3.49 ± 0.18 b	4.38 ± 0.43 ab	4.63 ± 0.48 a
**Malvidin-3-*O*-acetilglucoside**	17.70 ± 0.63 e	32.95 ± 0.13 b	32.42 ± 0.3 b	22.52 ± 0.92 d	37.21 ± 0.93 a	29.19 ± 0.23 c
**Peonidin-3-*O*-coumarylglucoside**	4.90 ± 0.07 d	4.68 ± 0.45 d	8.28 ± 1.70 bc	9.48 ± 0.58 b	11.87 ± 1.09 a	7.71 ± 0.51 c
**Malvidin-3-*O*-coumarylglucoside**	48.88 ± 0.87 e	84.12 ± 2.01 b	81.25 ± 4.88 bc	65.27 ± 6.89 d	100.62 ± 1.35 a	76.77 ± 1.49 c
**Total detected anthocyanins**	**179.19 ± 3.94 e**	**348.16 ± 6.96 b**	**334.60 ± 8.10 c**	**271.38 ± 9.94 d**	**402.43 ± 6.49 a**	**328.11 ± 5.30 c**
**Total detected phenolic compounds**	**334.96 ± 9.97 d**	**494.58 ± 11.29 b**	**506.01 ± 10.17 b**	**472.15 ± 15.84 c**	**582.04 ± 9.61 a**	**595.83 ± 9.07 a**

Each value represents the mean ± standard deviation, with n = 3. Significant differences at the *p* ≤ 0.05 level (LSD test) are indicated with lowercase letters. Treatments: control—standard 7-day maceration (K7); pre-fermentative cryomaceration (8 °C, 48 h) followed by a 13-day (C15) and 28-day (C30) period of simultaneous fermentation/maceration and extended post-fermentative maceration; pre-fermentative cryomaceration (8 °C, 48 h) followed by saignée procedure, and 13-day period of simultaneous fermentation/maceration and extended post-fermentative maceration (CS15); pre-fermentative hot maceration at (50 °C, 48-h) followed by a 13-day (H15) and 28-day (H30) period of simultaneous fermentation/maceration and extended post-fermentative maceration.

The obtained results showed that the total concentration of detected phenols was significantly higher in wine lees of all the treatments in comparison to the control treatment (K7), where 334.96 mg/100 g DW was determined. Significantly, the highest concentrations, 582.04 mg/100 g DW in the C30 treatment and 595.83 mg/100 g DW in the H30 treatment, were obtained by treatments where a 30-day extended maceration was conducted, regardless of the pre-fermentative procedure, i.e., cooling or heating. This outcome was in accordance with the fact that wine lees are a residual precipitate formed during wine fermentation and maceration, respectively; the longer the maceration period, the more it will precipitate [57].

In the studies published by [51,58], it was reported that a significant fraction of the anthocyanins extracted from grape skins can be adsorbed by yeasts and will precipitate in the lees. Consequently, the most abundant group of phenolic compounds found in Teran wine lees were indeed anthocyanins (Figure 1, Table 4), with the maximum total concentration reached in the C30 treatment with pre-fermentative mash cooling and 30-day maceration, following with the saignée treatment (CS15), where substantial quantities were also found. When comparing pre-fermentative procedures, cooling or heating, within a particular maceration duration, 15 or 30 days, higher concentrations of anthocyanins were found in treatments with cooling, C15 or C30, in comparison to their counterparts that included heating, H15 or H30, respectively. Consequently, the results regarding the precipitation of anthocyanins into wine lees suggested that pre-fermentative heating had more influence on the extraction of these compounds from solid parts of the grape, which was evident when observing results in grape skins, but also had influence on their interconversions and stability, which could have reflected their lower tendency to bind with lees and precipitate.

Considering flavan-3-ols, a significantly higher concentration was obtained in treatments with a 30-day maceration (C30 and H30) in comparison to other treatments. When comparing pre-fermentative procedures, cooling or heating, within particular maceration durations, 15 and 30 days, pre-fermentative heating (H15 or H30) had a stronger effect on the sedimentation of flavan-3-ols in wine lees in relation to pre-fermentative cooling (C15 or C30). The concentrations of flavan-3-ols aligned well to those found in the corresponding wines from the same experiment [29], suggesting the extent of their extraction from grape seeds reflected similarly on their contents in both wines and wine lees. It appeared that length of maceration had a more profound effect on the precipitation of flavan-3-ols than the pre-fermentative mash procedure. In the case of HC acids, pre-fermentative heating caused the stronger precipitation of HC acids, with the highest concentration found in the 30-day maceration treatment (H30), followed by the H15 treatment with the 15-day maceration.

Significantly, the highest concentrations of total detected HB acids and stilbenes were reached in wine lees with the pre-fermentative cooling treatment and 15-day maceration (C15) (Table 4). The result of HB acids corresponded to a similar outcome found for grape skins (Table 2). It is clear that the extent of the two competitive phenomena, i.e., the diffusion of these compounds from grape skins and their proneness to adsorption onto yeasts and solids followed by precipitation, together with the impact of the applied vinification technologies and other factors, shaped their distribution in various vinification products in a complex manner.

In the case of flavonols, the effect of pre-fermentative heating was more pronounced, despite the maceration duration (H15 and H30). Although flavonols are indeed known for their role in copigmentation interactions with anthocyanidins [25,59], pre-fermentative heating might reduce the interaction between anthocyanins and other phenolic compounds, such as flavonols, probably leading to a reduced extent of copigmentation, which leave flavonols unbound and to be subsequently precipitated in wine lees in greater amounts in the heating treatments (H15 and H30). In corresponding Teran wines, pre-fermentative heating in treatments H15 and H30 led also to a higher amount of flavonols, possibly due to the complex interactions during extraction, and subsequently higher quantity of flavonols available for sedimentation in lees [29]. Additionally, regarding grape seeds, interestingly, the C30 treatment had lower flavanol concentrations than C15 and H15 treatments as well, again pointing to the complex nature of the transfer of phenols between various phases and products of vinification.

### 3.4. Total Phenolic Content and Antioxidant Activity of Grape Skin, Seed, and Wine Lees Extracts

**Figure 2:**
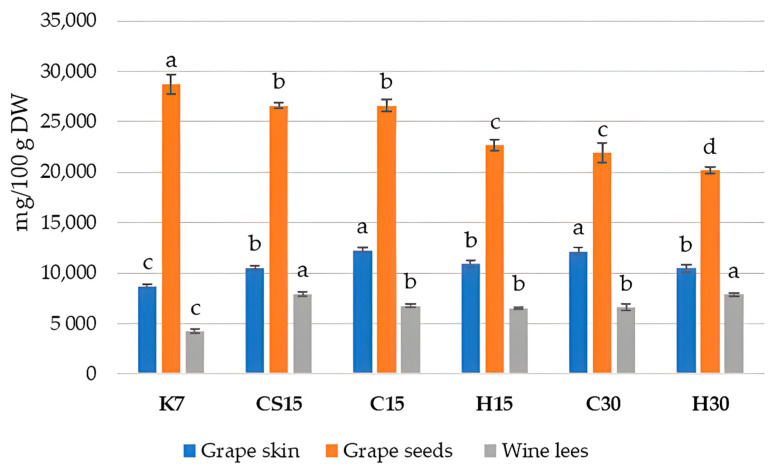
Total phenolic content in grape skin, seed, and wine lees samples *cv*. Teran (mg GAE/100 g DW). Each value represents the mean ± standard deviation, with n = 3. Significant differences at the *p* ≤ 0.05 level (LSD test) are indicated with lowercase letters. Treatments: control—standard 7-day maceration (K7); pre-fermentative cryomaceration (8 °C, 48 h) followed by a 13-day (C15) and 28-day (C30) period of simultaneous fermentation/maceration and extended post-fermentative maceration; pre-fermentative cryomaceration (8 °C, 48 h) followed by saignée procedure, and 13-day period of simultaneous fermentation/maceration and extended post-fermentative maceration (CS15); pre-fermentative hot maceration at (50 °C, 48-h) followed by a 13-day (H15) and 28-day (H30) period of simultaneous fermentation/maceration and extended post-fermentative maceration.

The obtained values of the spectrophotometric analysis of the total phenolic content (TPC) in Teran by-products are reported in Figure 2.

The presented results showed that the TPC found in Teran grape skins and wine lees of all the treatments was significantly higher compared to that in the control treatment (K7). Given that 7 days of maceration is a relatively short period, it is likely that the re-adsorption of phenolic compounds from other solid components, especially from seeds, back into the grape skins does not occur significantly, as might be the case in other treatments with prolonged macerations. Hence, the total phenolic content in the grape skins of the K7 treatment remained the lowest, as well in wine lees, while the TPC in grape seeds was the highest, consequently leading to wines with the lowest TPC in K7, as reported in a previous study on Teran wine from the same experiment [33]. It was reported that the total phenol content from skins was rapidly extracted within 4–5 days, whereas that from seeds was extracted progressively. Thus, the difference in the tissue structure between berry skins and seeds probably causes the difference in the phenolic extraction between them [60,61]. The obtained results once again point to the complexity of the transformations and transitions of diverse classes of phenolic compounds between various systems during vinification, especially when affected by specific winemaking technologies.

When observing the TPC in Teran grape skin samples, it ranged from 8692.66 mg GAE/100 g DW (K7) to 12,252.86 mg GAE/100 g DW (C15), which is a considerably high phenol content, in comparison to some other varieties like the Primitivo variety, with a TPC of 1328 mg CE/100 g DW [42], or the Cabernet Sauvignon and Merlot variety, with 7475 mg GAE/100 g DW and 4623 mg GAE/100 g DW, respectively [46]. Significantly, the most elevated content in Teran skin samples was obtained in treatments C15 and C30, suggesting pre-fermentative cooling had the most significant impact, regardless of maceration duration. Such a result did not fully coincide with the results of the HPLC analysis of phenols (Table 2), possibly because of the significant contribution of proanthocyanidins (condensed tannins) which were not analyzed by HPLC. It seems that pre-fermentative cooling preserves most phenols in grape skins when compared to other treatments, probably due to the fact that lower temperatures reduce their solubility in the liquid phase.

Regarding the TPC values determined in grape seed samples (Figure 2), the results agreed with those determined by HPLC (Table 3). Statistically, the highest content was detected in the control treatment (K7), which was 28,733.55 mg GAE/100 g DW. The TPC values determined in other treatments ranged from 20,187.54 mg GAE/100 g DW in H30 to 26,593.67 mg GAE/100 g DW in CS15. The obtained results in Teran seed samples were quite higher in comparison with other findings [50], where Pinot Noir contained 16,518 mg CE/100 g DW, and Marselan grape seeds contained 11,122 mg GAE/100 g DW.

Such results confirmed that the applied pre-fermentative and post-fermentative vinification technologies in other treatments (CS15, C15, H15, C30 and H30) strongly favored the extraction of phenols from seeds into wine, leaving the highest quantities in K7 seeds. When observing differences between particular pre-fermentative mash procedures, i.e., cooling or heating, lower TPC values were found in treatments with the longer 30-day maceration duration (C30 or H30), confirming once again that extended maceration in the presence of ethanol enhances the extraction of phenols from seeds due to the increased permeability of their tissues [12,39]. Moreover, the effect of heating also had a notable impact on the extraction of phenols within particular treatments submitted to 15 (H15) or 30 days (H30) of prolonged maceration in comparison to cooling treatments (C15 or C30).

In Teran wine lees, TPC values ranged from 4230.23 to 7926.60 mg GAE/100 g DW; the most statistically increased levels were demonstrated in the CS15 treatment, where the saignée procedure was performed, and in the H30 treatment with the pre-fermentative hot maceration and extended maceration of 30 days. When considering the other literature data, for example in Blatina wine lees [57], the TPC was found to be 2316.6 ± 37.9 mg GAE/100 g dry weight (DW), which is 1.8 to 3.4-fold lower when related to our findings. The lowest value was determined in the control (K7) wine lees, as a consequence of a lower degree of phenol extraction and, consequently, lower amounts of phenols available for precipitation during and at the end of fermentation. The obtained results made it evident that pre-fermentative mash procedures, such as cooling, heating, and the saignée procedure, along with prolonged post-fermentative maceration, may have a great impact on the integrity and permeability of cell walls, thereby modulating the availability of chemical compounds for extraction, as it was also shown previously [40,62,63].

**Figure 3:**
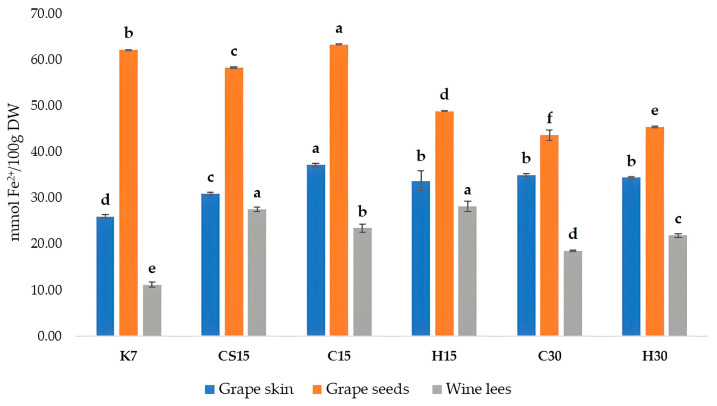
Antioxidant activity, determined by FRAP assay in grape skin, seed, and wine lees samples of *cv*. Teran (mmol Fe^2+^/100 g DW). Each value represents the mean ± standard deviation, with n = 3. Significant differences at the *p* ≤ 0.05 level (LSD test) are indicated with lowercase letters. Treatments: control—standard 7-day maceration (K7); pre-fermentative cryomaceration (8 °C, 48 h) followed by a 13-day (C15) and 28-day (C30) period of simultaneous fermentation/maceration and extended post-fermentative maceration; pre-fermentative cryomaceration (8 °C, 48 h) followed by saignée procedure, and 13-day period of simultaneous fermentation/maceration and extended post-fermentative maceration (CS15); pre-fermentative hot maceration at (50 °C, 48-h) followed by a 13-day (H15) and 28-day (H30) period of simultaneous fermentation/maceration and extended post-fermentative maceration.

When considering the antioxidant activity of Teran by-products, significant differences between the treatments were also observed (Figure 3). Antioxidant activity was measured by a colorimetric method called the FRAP (ferric reducing/antioxidant power) assay, which has the ability to reveal compounds capable of reducing Fe^3^⁺ to Fe^2^⁺. Antioxidant activity, i.e., the reducing ability of grape skins and wine lees of all the treatments, was significantly higher in comparison to control treatment (K7). The FRAP values measured in grape skin ranged from 25.94 (K7) to 37.18 mM Fe^2+^/100 g DW (C15), in grape seeds from 43.62 (C30) to 62.10 mM Fe^2+^/100 g DW (K7), and in wine lees from 11.14 (K7) to 28.18 mM Fe^2+^/100 g DW (H15). When comparing to other investigations, a group of authors [12] obtained slightly lower results in comparison to our findings, with FRAP values for crude seed extracts ranging from 19.3 to 26.75 mM Fe^2+^/100 g DW, and for crude skin extracts varied from 10.53 to 26.68 mM Fe^2+^/100 g DW, depending on the red grape variety. Regarding the antioxidant activity of wine lees samples, our results were lower in comparison to an investigation on the Blatina variety [57], which reported a result of 45.70 mM of Trolox equivalents per 100 g of dry mass.

The correlation analysis exposed a strong, positive correlation between FRAP and TPC values, for all three by-products, with correlation coefficients (r) for seeds, r = 0.89, skins r = 0.93, and wine lees r = 0.77, respectively. Such correlations confirmed that phenolic compounds are the primary contributors to the antioxidant activity of extracts obtained from wine by-products, as stated previously [64]. The antioxidant activity values for skins and seeds found in this study were slightly higher than those reported by [12].

In grape skins and seeds, the highest antioxidant activity was determined in the C15 treatment, where pre-fermentative cooling and a 15-day maceration was performed. This finding about grape seeds is interesting, since the most increased total phenolic content and concentration of all detected phenolic compounds by HPLC were determined in control treatment K7. Such an apparent discrepancy possibly arose from different redox potentials and structural characteristics of various individual phenolic compounds, including the degree of hydroxylation and the extent of conjugation [42,65], meaning that particular phenol classes could have had a stronger impact on the antioxidant activity despite their lower absolute concentrations, and vice versa.

Regarding wine lees, treatments subjected to a 15-day maceration (CS15, C15, and H15) had higher antioxidant activity values than treatments subjected to a 30-day maceration, regardless of the pre-fermentative mash procedure (cooling or heating), roughly suggesting that compounds extracted during the 15-day prolonged maceration had stronger antioxidant capacity in comparison to those from the 30-day maceration treatments.

### 3.5. Principal Component Analysis

To better visualize the differences between by-products obtained upon the pre-fermentative and post-fermentative vinification technologies applied during the vinification of a Teran red variety based on bioactive phenols determined by HPLC, total phenolic content, and antioxidant activity, an unsupervised statistical analysis using PCA was performed separately for each vinification by-product, that is, grape skins, grape seeds, and wine lees.

**Figure 4:**
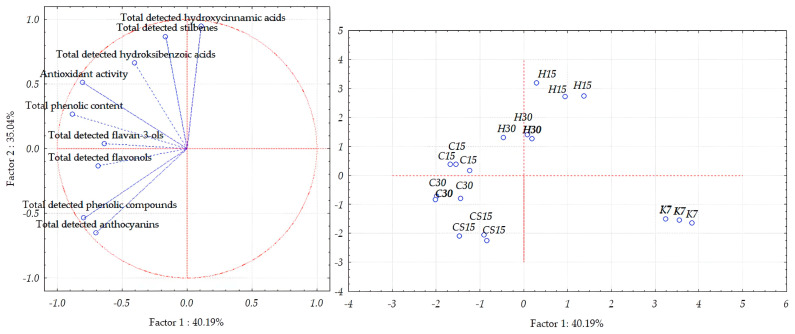
Separation of Teran grape skin samples obtained after application of pre-fermentative and post-fermentative vinification technologies presented in three replications in two-dimensional space defined by the first two principal components (PC1 and PC2) based on the concentrations of bioactive phenolic compounds and antioxidant activity. Treatments: control—standard 7-day maceration (K7); pre-fermentative cryomaceration (8 °C, 48 h) followed by a 13-day (C15) and 28-day (C30) period of simultaneous fermentation/maceration and extended post-fermentative maceration; pre-fermentative cryomaceration (8 °C, 48 h) followed by saignée procedure, and 13-day period of simultaneous fermentation/maceration and extended post-fermentative maceration (CS15); pre-fermentative hot maceration at (50 °C, 48-h) followed by a 13-day (H15) and 28-day (H30) period of simultaneous fermentation/maceration and extended post-fermentative maceration.

Concerning the separation of Teran grape skin samples, the first two principal components, PC1 and PC2, explained 75.23% of the total variance (Figure 4), thus enabling a good separation of grape skin samples obtained by different treatments. The first principal component accounted for 40.19% of the total variance, whereas the second principal component (PC2) accounted for 35.04%.

**Figure 5:**
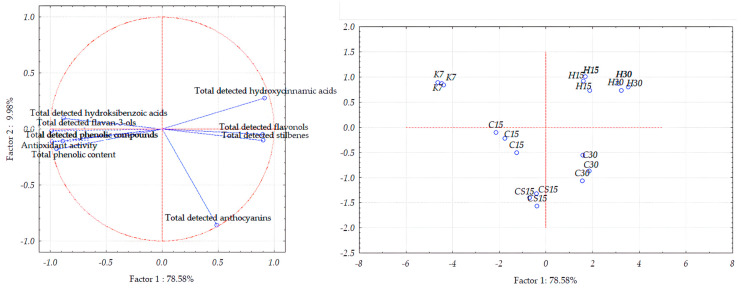
Separation of Teran grape seed samples obtained after application of pre-fermentative and post-fermentative vinification technologies presented in three replications in two-dimensional space defined by the first two principal components (PC1 and PC2) based on the concentrations of bioactive phenolic compounds and antioxidant activity. Treatments: control—standard 7-day maceration (K7); pre-fermentative cryomaceration (8 °C, 48 h) followed by a 13-day (C15) and 28-day (C30) period of simultaneous fermentation/maceration and extended post-fermentative maceration; pre-fermentative cryomaceration (8 °C, 48 h) followed by saignée procedure, and 13-day period of simultaneous fermentation/maceration and extended post-fermentative maceration (CS15); pre-fermentative hot maceration at (50 °C, 48-h) followed by a 13-day (H15) and 28-day (H30) period of simultaneous fermentation/maceration and extended post-fermentative maceration.

In the case of Teran grape seed samples, the first two principal components accounted for 88.53% of the total variance (Figure 5). However, the distribution between the two components differs slightly, with PC1 explaining 78.58% and PC2 accounting for 9.98%.

**Figure 6:**
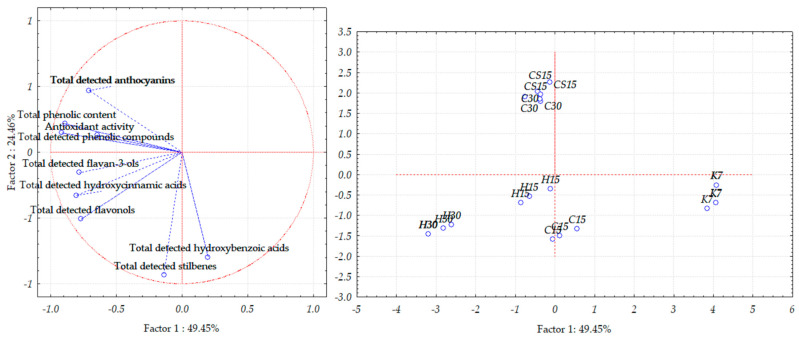
Separation of Teran wine lees samples obtained after application of pre-fermentative and post-fermentative vinification technologies presented in three replications in two-dimensional space defined by the first two principal components (PC1 and PC2) based on the concentrations of bioactive phenolic compounds and antioxidant activity. Treatments: control—standard 7-day maceration (K7); pre-fermentative cryomaceration (8 °C, 48 h) followed by a 13-day (C15) and 28-day (C30) period of simultaneous fermentation/maceration and extended post-fermentative maceration; pre-fermentative cryomaceration (8 °C, 48 h) followed by saignée procedure, and 13-day period of simultaneous fermentation/maceration and extended post-fermentative maceration (CS15); pre-fermentative hot maceration at (50 °C, 48-h) followed by a 13-day (H15) and 28-day (H30) period of simultaneous fermentation/maceration and extended post-fermentative maceration.

When observing Teran wine lees samples, PCA allowed for a good separation of the investigated samples, as the first two principal components, PC1 and PC2, explained 73.91% of the total variance (Figure 6); more precisely, the first component demonstrated 49.45% of the total variance, and the second one demonstrated 24.46% of the total variance.

In PCA plots which showed the separation of grape skins and wine lees samples (Figure 4 and Figure 5), the control treatment (K7) was distinctly differentiated from the others, mostly along PC1 but also along PC2, thus indicating the weakest correlation of the control K7 treatment with the quantities of all the analyzed compounds and features among the treatments. These results align with the results of the one-way ANOVA reported in Table 2 and Table 3 and Figure 1, Figure 2 and Figure 3, since the samples from the K7 treatment exhibited the lowest concentration of the majority of detected phenolic compounds, as well as the lowest value of the TPC and antioxidant activity.

When observing the PCA plot separating grape skin samples (Figure 4), it is evident that treatments C15, C30, and CS15 were mostly correlated with total detected phenolic compounds, anthocyanins, flavan-3-ols, and flavonols, as well as total phenolic content, total antioxidant activity, and total detected hydroxybenzoic acids. On the opposite side of the Cartesian coordinate system with respect to the PC1 axis, the H15 treatment correlated notably with total detected hydroxycinnamic acids and stilbenes, and to a lesser extent with total phenolic content, total antioxidant activity, and total detected hydroxybenzoic acids. When observing the entire grape skin PCA plot, it is evident that a significant number of detected phenolic compounds correlated more with the treatments submitted to cryomaceration (CS15, C15, and C30). Such a relation could be compared with the results of the PCA for Teran wines obtained by the same treatments [29], where oppositely, the majority of detected compounds corelated more with H15 and H30 treatment wines. It could be generally confirmed that heating treatments enhanced the extraction of phenolic compounds into wine, whereas in grape skin samples, the cooling procedure did not boost extraction to the same extent as heating, therefore leaving more phenolic compounds retained in the grape skin structures.

Furthermore, regarding grape seed samples (Figure 5), total detected phenolic compounds, hydroxybenzoic acids, and flavan-3-ols determined by HPLC, total phenolic content, and antioxidant activity showed the strongest correlation with K7 and C15 treatments. These results were consistent with those obtained by the one-way ANOVA (Table 3, Figure 1, Figure 2 and Figure 3), emphasizing the strong effect of pre-fermentative and post-fermentative vinification technologies on the extraction of phenolic compounds from grape seeds to wine, which left the highest concentration of phenolic compounds retained in the seeds of the control treatment (K7). The PCA allowed a good separation of investigated seed samples along the first principal component, since treatments C15 and CS15 were clearly separated from C30, H15, and H30, which is also evident with the high percentage of the variance (PC1 78.58%). The former was correlated most with total detected hydroxybenzoic acids, flavan-3-ols, phenolic compounds, antioxidant activity, and total phenolic content, while the latter was mostly correlated with total detected hydroxycinnamic acids, flavonols, and stilbenes. The obtained results of the grape seed PCA analysis were in line with the results obtained for the Teran wine wherefrom these by-products originated, with the control wine being characterized by the significantly lowest concentrations of total detected hydroxybenzoic acids, flavan-3-ols, and the lowest total phenolic content and antioxidant activity [29], showing that the lesser extent of the extraction of mentioned compounds from grape seed was directly reflected to their content in wine samples.

On the other hand, when analyzing the wine lees PCA plot (Figure 6), it is evident that the total detected anthocyanins correlated with CS15 and C30 treatments, similar to the grape skin PCA plot (Figure 4). It is evident that the pre-fermentative mash cooling procedure increased the degree of retention of anthocyanin compounds in wine lees. This effect was also evident when observing the PCA plot of corresponding wines, where treatments submitted to pre-fermentative cooling (CS15, C15, and C30) did not correlate with total anthocyanins at all due to their precipitation in wine lees or retention on grape solids [29]. This can be explained by the fact that anthocyanins have reduced solubility in cooler conditions, making it likely that cryomaceration limits their solubility in the wine, leading to greater precipitation into the wine lees. The weakest separation was shown in the case of wine lees as the majority of treatments (CS15, C30, C15, and H15) mostly aligned on the interception of the two axes, which indicates a slightly lower correlation with analyzed variables. The lowest correlation was observed for the K7 treatment, as it was positioned entirely on the opposite side of the Cartesian system. The highest correlation with observed variables was noted in the case of the H30 treatment. Regarding the precipitation of flavan-3-ols, hydroxycinnamic acids, and flavonols into wine lees, the effect of pre-fermentative mash heating in treatments (H15 and H30) was clearly noticeable, particularly when observing the PCA plot.

## 4. Conclusions

The results indicate that Teran red wine by-products, such as grape skins, seeds, and wine lees, are an abundant source of phenolic compounds with strong bioactive properties that provide added value to by-products, which is especially important when viewed in the context of circular economy. This study highlighted a notable impact of pre-fermentative and post-fermentative vinification technologies on grape skin and wine lees samples, showing significantly higher total concentrations of individual phenolic compounds, antioxidant value, and total phenolic content in comparison to the control. The total amount of individual phenolic compounds and anthocyanins in grape skins were under the strong impact of pre-fermentative cooling, with the highest concentrations found in the saignée treatment (CS15). Prolonged 15-day macerations, with both pre-fermentative cooling and heating (C15 and H15), preserved more hydroxybenzoic acids, while the effect of pre-fermentative heating was most evident in the case of hydroxycinammic acid and stilbenes. Regarding grape seeds, the prolonged 30-day maceration strongly reduced the levels of flavan-3-ols, total phenolic content, and antioxidant activity, indicating the exhaustion of the seeds by soaking in an ethanol-rich medium. The greatest effect on the precipitation of total detected phenolic compounds in wine lees was the extended 30-day maceration, where the highest concentration was observed despite the applied pre-fermentative mash procedure (C30 and H30). The valorization of winery by-products presents a promising, sustainable approach in the winemaking industry, by converting waste into valuable resources, thus promoting environmental responsibility, and acceptability for utilization in various industries for developing novel products. The specific significance of this research lies in the evaluation of wine by-products, grape skins, seeds, and wine lees of Teran, the most widely cultivated autochthonous red grape variety in Istria.

## Figures and Tables

**Table 1:** Experimental design of Teran red wine vinification.

Treatment	Pre-Fermentative Maceration Procedure		Fermentation and Maceration			Pre-Fermentative Maceration Procedure + Maceration Duration
			Vinification Technology—Maceration	Fermentation/Maceration Temperature	Maceration Duration	
**K7**	/		Standard maceration	24 °C	7 days	/
**CS15**	Cooling at 8 °C, 48 h (cryomaceration)	Saignée	Fermentation/maceration + prolonged post-fermentative maceration		13 days	15 days
**C15**					13 days	15 days
**C30**					28 days	30 days
**H15**	Heating at 50 °C, 48 h (hot pre-fermentative maceration)				13 days	15 days
**H30**					28 days	30 days

Abbreviations: K—control; C—cooling; H—heating; S—saignée; 7, 15, and 30 represent the total durations of maceration in days.

## Data Availability

The original contributions presented in the study are included in the article/Supplementary Material, further inquiries can be directed to the corresponding author.

## Supplementary Materials

The following supporting information can be downloaded at: https://www.mdpi.com/article/10.3390/foods13213493/s1, Table S1: Parameters of method: compound standard used, retention time, detection wavelengths, calibration equation, coefficient of determination; Table S2: Parameters of method: standard solution, calibration equation, and coefficient of determination.
