# Supplementary material for: Evaluating the Impact of Pre-Fermentative and Post-Fermentative Vinification Technologies on Bioactive Compounds and Antioxidant Activity of Teran Red Wine By-Products
Source: Foods. 2024 Oct 31;13(21):3493. doi: 10.3390/foods13213493 (PMC11545498; doi:10.3390/foods13213493)
Supplement: Supplementary file 1 [file foods-13-03493-s001.zip › foods-3272733-supplementary.pdf]

Supplementary material and data

Table S1: Parameters of method: compound standard used, retention time, detection wavelengths, calibration equitation, and coefficient of determination

Phenolic compound	Compound standard used	Retention time (min) of standard	DAD λ/nm	FLD λ_{ex}/nm; FLD λ_{em}/nm	Calibration equation	Coefficient of determination (R^2)
**Anthocyanins**						
Delphinidin-3-*O*-glucoside	Delphinidin-3-glucoside chloride	12.5	518 nm		y = 59.67532x - 13.44297	R^2 = 0.99858
Cyanidin-3-*O*-glucoside	Cyanidin-3-glucoside chloride	14.3	518 nm		y = 128.00411x - 0.37337	R^2 = 0.99994
Petunidin-3-*O*-glucoside	Petunidin-3-glucoside chloride	15.6	518 nm		y = 101.27391x - 10.24165	R^2 = 0.99997
Peonidin-3-*O*-glucoside	Peonidin-3-glucoside chloride	18.3	518 nm		y = 96.14081x + 0.11739	R^2 = 0.99996
Malvidin-3-*O*-glucoside	Malvidin-3-glucoside chloride	19.8	518 nm		y = 50.13246x - 0.28033	R^2 = 0.99995
Peonidin-3-*O*-acetylglucoside	Peonidin-3-glucoside chloride		518 nm		y = 96.14081x + 0.11739	R^2 = 0.99996
Malvidin-3-*O*-acetylglucoside	Malvidin-3-glucoside chloride		518 nm		y = 50.13246x - 0.28033	R^2 = 0.99995
Peonidin-3-*O*-cumarylglucoside	Peonidin-3-glucoside chloride		518 nm		y = 96.14081x + 0.11739	R^2 = 0.99996
Malvidin-3-*O*-cumarylglucoside	Malvidin-3-glucoside chloride		518 nm		y = 50.13246x - 0.28033	R^2 = 0.99995
**Phenolic acids**						
Gallic acid	Gallic acid	7.4	280 nm		y = 56.165x + 1.931	R^2 = 0.9996
Protocatechuic acid	Protocatechuic acid	10.3	280 nm		y = 33.508x - 0.295	R^2 = 1.0000
Vanilic acid	Vanillic acid	15.3	280 nm		y = 39.823x - 0.2313	R^2 = 1.0000
Syringic acid	Syringic acid	16.8	280 nm		y = 60.652x - 1.3293	R^2 = 0.9998
trans-Caftaric acid	*trans*-Caftaric acid	12.3	330 nm		y = 65.726x - 1.3289	R^2 = 1.0000
**Flavonols**						
Quercetin-3-O-glucoside	Quercetin-3-O-glucoside	27.1	360 nm		y = 98.201x - 2.4772	R^2 = 0.9994
Quercetin	Quercetin hydrate	46.6	360 nm		y = 85.352x - 16.025	R^2 = 0.9999
Kaempferol	Kaempferol	49.3	360 nm		y = 84.106x - 2.5405	R^2 = 0.999
**Flavan-3-ols**						
Procyanidin B1	Procyanidin B1	11.8		280 nm; 320 nm	y = 74.578x - 8.6541	R^2 = 0.9999
Procyanidin B3	Procyanidin B3	12.5		280 nm; 320 nm	y = 73.05x - 8.9826	R^2 = 0.9998

(+)-Catechin	(+)-Catechin hydrate	13.1		280 nm; 320 nm	y = 168.97x - 2.8262	R^2 = 1.0000
Procyanidin B2	Procyanidin B2	14.7		280 nm; 320 nm	y = 76.883x - 12.038	R^2 = 0.9993
(-)-Epicatechin	(-)-Epicatechin	16.3		280 nm; 320 nm	y = 125.49x - 4.3968	R^2 = 1.0000
Procyanidin C1	Procyanidin C1	18.1		280 nm; 320 nm	y = 152.64x - 7.0295	R^2 = 0.9987
**Stilbenes**						
trans-Piceid	Polydatin (*trans*-piceid)	23.7	306 nm		y = 97.763x - 1.1117	R^2 = 0.9997
trans-Resveratrol	*trans*-Resveratrol	39.0	306 nm		y = 108.78x - 11.521	R^2 = 0.9978

Table S2: Parameters of method: standard solution, calibration equation, and coefficient of determination

**Method**	**Standard solution**	**Calibration equation**	**Coefficient of determination (R^2)**
Ferric reducing/antioxidant power assay (FRAP)	$FeSO_4$ x $7H_2O$	y = 2.0357x - 0.0204	R^2 = 0.9989
Total phenolic content (TPC)	Gallic acid monohydrate	y = 0.0014x - 0.0213	R^2 = 1.0000
